# Characterization and Applications of Colloidal Systems as Versatile Drug Delivery Carriers for Parenteral Formulations

**DOI:** 10.3390/ph14020108

**Published:** 2021-01-29

**Authors:** Lakshmi Prasanna Kolluru, Prachi Atre, Syed A. A. Rizvi

**Affiliations:** 1 Vaccine Nanotechnology Laboratory, College of Pharmacy, Mercer University, 3001 Mercer University Drive, Atlanta, GA 30341, USA; kolluruprasu@gmail.com; 2 Capstone Development Services, Rosemont, IL 60018, USA; 3 College of Pharmacy and Health Sciences, St. John’s University, Queens, NY 11439, USA; prachiatre.24@gmail.com; 4 School of Pharmacy, Hampton University, Hampton, VA 23668, USA

**Keywords:** drug delivery, natural surfactants, self-assembled systems, drug solubility

## Abstract

Preparing a suitable formulation for parenteral administration is already a difficult task; this, coupled with poor water-soluble new chemical entity (NCE), complicates this situation even further. There are several methodologies available to enhance water solubility, but this alone does not entail successful formulation. Making a micro/nano emulsion with a suitable surfactant not only increases the drug solubility but also the cell membrane permeability. Thus, not only biopharmaceutic classification system (BCS)-II (low solubility compounds) but also BCS-III (low permeability) and BCS-IV drugs (low solubility and low permeability) can be further exploited. Those drug candidates otherwise will not move further in NCE evaluation or clinical trials. This succinct review article delves into various aspects of biphasic micro/nano emulsion systems for parenteral drug delivery including the structure of the biphasic colloidal systems, characterization parameters, stability issues, regulatory considerations, and applications in life sciences.

## 1. Introduction

Poorly water-soluble molecules are a challenging problem in the development of suitable pharmaceutical drug formulation. This situation gets further complicated by the fact that most of the newly developed drugs exhibit poor solubility in organic media as well. Consequently, erratic absorption characteristics and low systemic bioavailability are typical issues with poorly water-soluble drugs [1,2]. The parenteral route of administration (viz. intravenous, intradermal, intramuscular, intraarterial, subcutaneous etc.), offers significantly high absorption profile and hence enhanced bioavailability [3]. Owing to low solubility of drugs, it is impossible and actually dangerous to administer solutions intravenously since it can potentially precipitate and clog the vessel [2]. Drug delivery scientists have used various formulation approaches to deal with problems associated with the delivery of hydrophobic drugs via the parenteral route [3]. The term parenteral is coined out of two words that are Greek in origin, viz. “para” meaning besides and “enteron” meaning gut. Thus, routes of drug administration that bypass the gastrointestinal tract are referred to as parenteral routes of drug delivery. The most critical method of drug delivery is the I.V. (intravenous) route and widely used heterogeneous systems for this route are simple oil-in-water (*o*/*w*) emulsions and multiple water-in-oil-in-water (*w*/*o*/*w*) formulations [4].

The traditional, and most common, approaches for parenteral delivery of poorly soluble drugs involve complexation, solubilization of hydrophobic agents in micelles and liposomes as drug carrier systems, among a few others. Although the aforementioned approaches are used for hydrophobic drug delivery, they have several limitations hindering the employment of their full potential. Cyclodextrins are expensive and may exhibit poor complexation with drug under consideration, limited micellar solubilization capacity and complexity coupled with the high cost of the manufacturing process of liposomes [3]. Therefore, there is a growing need to improve formulation strategies to improve the parenteral delivery of hydrophobic drugs.

In the pharmaceutical arena, emulsions as well as micro-emulsions are widely accepted carriers for the delivery of both lipophilic (hydrophobic) and lipophobic drugs, including the ones with low permeability. Lately, micro-nano-emulsions have acquired increased focus in pharma applications as drug carriers [4], since they have great potential to deal with problems that are related to drug delivery of poorly water and also lipid soluble drugs [5]. This brief and succinct review focuses on the diverse aspects of submicron emulsions and nano-suspensions including the structure of colloidal systems, scientific and regulatory considerations in development, FDA approved colloidal systems for parenteral delivery and key characterization techniques needed for the successful approval of these colloidal systems. Finally, the application of these carrier systems as promising formulation approaches in parenteral drug delivery, including Total Parenteral Nutrition, Vaccine Delivery, Long-Acting Injectable Therapy, and Anti-Cancer Drugs and Diagnostic Agents, will also be highlighted.

## 2. Discussion

### 2.1. Necessity and Potential of Biphasic Colloidal Carriers in Parenteral Drug Delivery

The usage of oil in water (*o*/*w*) emulsion by incorporation of the drug in oil phase (dispersed phase) can help in reducing the problems associated with hydrophobic drugs. Furthermore, a submicron emulsion can be a substitute to a co-solvent based formulation that generally results in drug precipitation at the site of administration [6]. Submicron emulsions are potential drug carrier systems with the following advantages over other dosage forms: (a) enhanced drug solubilization and bioavailability; (b) require minimum amount of energy for formation and are thermodynamically stable systems; (c) targeted and controlled release colloidal drug delivery systems; (d) drug incorporation in non-polar phase in o/w micro-emulsion protects drugs that are susceptible to hydrolysis and oxidation; (e) aqueous dosage form for hydrophobic drugs [7]. Micro-emulsions have demonstrated their potential as commercially feasible colloidal drug carrier systems for parenteral delivery. With appropriate selection of excipients, a parenteral micro-emulsion can be formulated with desired attributes such as sustained release and extended circulation in blood [3]. Drug loading into the dispersed phase can allow sustained and/or prolonged release depending on the hydrophobicity of the active ingredient. Hydrophobic and hydrophilic drugs can be incorporated into the dispersed phase of *o*/*w* and w/o emulsion systems, respectively [6]. Rhee et al. developed an o/w parenteral micro-emulsion system containing itraconazole. A mixture of medium chain triglyceride and benzyl alcohol was chosen as dispersed (oil) phase. The mean droplet size of the micro-emulsion formulation was <150 nm. A comparison of pharmacokinetic profiles of itraconazole and its metabolite hydroxyitraconazole of itraconazole micro-emulsion with PEG 400 solution and cyclodextrin formulation highlighted the potential of the micro-emulsion system [8]. Nesamony et al. demonstrated the development of *w*/*o* emulsion using ethyl oleate (EO), dioctyl sodium sulfosuccinate (DOSS) and water. Further, prepared formulations were characterized using (polarized) light microscopy, electrical conductivity, rheology, and dynamic light scattering. In addition, aseptic filtration as mode of sterilization and in-vitro cell toxicity studies revealed the potential of formulated submicron emulsion [9].

Coarse solid suspensions have been manufactured for many years for parenteral administration by subcutaneous route (HUMULIN, LENTE) or intramuscular (Bicillin^®^ L-A) delivery. Lately, nanotechnology has been used to overcome low solubility and poor bioavailability problems. Besides, nanotechnology is also used to achieve targeted (site-specific) drug delivery. Parenteral nanosuspensions can reduce irritation and control the rate of drug administration. The affix nano is derived from a Greek word that means dwarf or small and was first used to denote any matter that is in the size range of the nanometer, and the term nanotechnology was first used by scientist Norio Taniguchi at the University of Tokyo, Japan.

Nanosuspensions have appeared as a promising drug delivery strategy for hydrophobic drugs via the parenteral route. Kolluru et al. has developed novel delivery system for the delivery of hard to solubilize Docetaxel based on polycaprolactone nanoparticle system stabilized with Pluronic F108 surfactant. This delivery system has not only enhanced solubility profile of the drug but also shown to be better localized than free drug in targeted delivery of drug to the tumor site. In addition, the slow release of the drug from the nanoparticles and nano size of the system will also reduce side effects and help to protect the drug from being rapidly cleared from the body [10]. Similarly, Etoposide-loaded bovine serum albumin (EPEG-BSA) nanosuspensions were formulated and characterized for in-vitro and in-vivo safety of the developed nanosuspension system. In contrast to Injection^®^, the formulated suspension showed a sustained drug release profile. Further, in vivo studies indicated reduced myelosuppression of EPEG in mice [11]. Finally, Tian et al. developed a *p*-terphenyl derivative (H2) nano-suspension by a combination of precipitation and micro-fluidization methods and later transformed it into dry powder by lyophilization. Marked enhancement was observed in dissolution rate with decreased particle size. In addition, the crystalline form of H2 was maintained after the particle size reduction process. Additionally, increased AUC and prolonged residence time revealed the potential of the H2 nanosuspension system [12].

### 2.2. Structure of Lipid-Based Biphasic Colloidal Systems

The concept of micro-emulsion was coined by Schulman and co-workers and it has been re-defined since then. Micro-emulsions are optically transparent, isotropic, low viscosity, thermodynamically stable dispersions of polar and non-polar phases stabilized by a combination of a surfactant and a co-surfactant. Nevertheless, structural microemulsions cannot only be considered as dispersions but rather a single percolated phase consisting of water or oil droplets, micelles or reverse micelles and bi-continuous structures that are characterized by the absence of internal or external phase [6,7].

**Figure 1:**
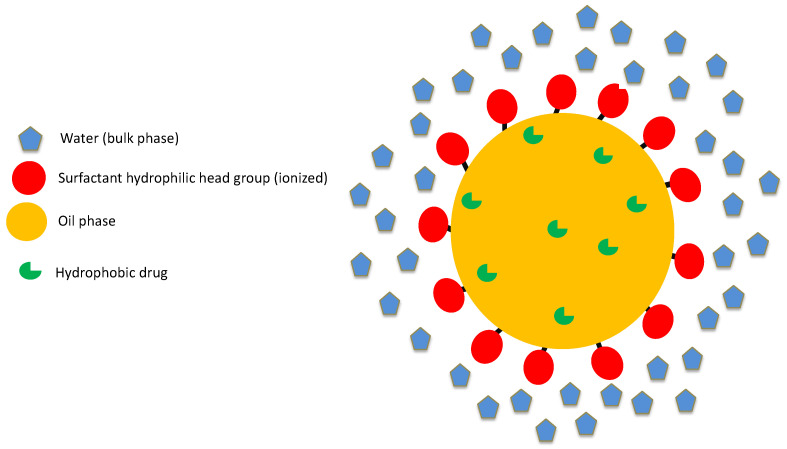
Structure of drug loaded emulsion system.

Emulsion can be defined as a dispersion of two immiscible liquids where one liquid is dispersed in the form of droplets/globules in the continuous phase of the other liquid. When the droplets/globules are of sub-micron size, the emulsion can be termed as micro/nano- emulsion. The two most used immiscible fluids are water and oil [13]. A submicron emulsion may contain considerable quantities of both oil and aqueous phases such that it seems to be a clear system but rather consists of submicroscopic dispersed regions that are oleic or aqueous in nature (Figure 1) [14]. Micellar emulsion is a dynamic system where the interface fluctuates continuously and spontaneously. Structural micro-emulsions are categorized as *w*/*o* systems wherein water droplets are dispersed in continuous non-polar (oil) phase, *o*/*w* systems in which oil globules are dispersed in continuous polar (aqueous) phase and bi continuous structures that contain similar amounts of oil and aqueous phases [15]. The mutual solubility of oil phase and aqueous phase is very low. However, with the addition of an amphiphile (surfactant), the solubility increases until a sufficiently high concentration of amphiphile is achieved, at which the mixture becomes homogeneous [16]. Pharmaceutical acceptance of excipients based on their toxicity makes the formulation of sub-micron emulsion critical.

Previous studies revealed that the self-micro-emulsifying process is specific to the nature and concentration of the surfactant and co-surfactant, surfactant/co-surfactant ratio, nature and ratio of the oil/surfactant pair and also the temperature at which emulsification occurs. Sub-micron emulsion formulation is a combination of three to five components: an oil (non-polar) phase, an aqueous (polar) phase, a surface-active agent, a secondary surfactant (co-surfactant) and sometimes an electrolyte [17]. The non-polar (oil) phase could be comprised of mono, di or triglycerol. The choice of an oil is critical as it affects the loading of the therapeutic active moiety, droplet size and physical and chemical properties that affect the stability of the micro-emulsion system [13]. The oil phase can solubilize the required dose of a hydrophobic drug and promote self-emulsification. For a successful micro-nano-emulsion formulation, both medium and long chain triglycerides (MCT and LCT), with varying degrees of saturation, can be used [18]. The oil component has an ability to penetrate and swell the non-polar (lipophilic) region of the surfactant monolayer, thereby influencing the curvature. Shorter chains tend to penetrate the lipophilic region of the surfactant more than longer chains, resulting in a negative curvature and smaller droplet size [17]. The surface-active agent selected must be able to reduce interfacial tension, provide a flexible film and provide pertinent hydrophobic characteristics to correct the curvature at the interface for the desired type of micro-emulsion. The four groups of surfactants used in the formulation of submicron emulsion are as follows [17,18]. (A) Anionic surfactants: the hydrophilic group carries a negative charge—these include sodium lauryl sulfate and potassium laurate; (B) cationic: the hydrophilic group carries a positive charge—these include quaternary ammonium halides; (C) non-ionic surfactants: the hydrophilic group carries no charge—these include Brij^®^35 and sorbitan monooleate; (D) zwitterionic/ampholytic: the hydrophilic group carries a positive as well as a negative charge—these include phospholipids and sulfobetaines. The combination of an ionic and a non-ionic surfactant is effectual in the formulation of micro-emulsion. HLB (Hydrophilic–Lipophilic Balance) of the surfactant is useful in the selection of the surfactant. The HLB takes into account the relative contributions of hydrophilic and lipophilic moieties in a surfactant. Surfactants with low HLB (3–6) favor formation of *w*/*o* submicron emulsions whereas high HLB surfactants (8–18) favor formation of *o*/*w* microemulsion systems [19].

### 2.3. Structure of Nanoparticulate Systems

For parenteral administration, the active moiety is either dissolved or has a particle/globule size <5 µm to avoid capillary blockade. Nanosuspensions are a unique approach for parenteral drug delivery. The absence of any toxic excipients viz. solvents/co-solvents makes the nanosuspension system tolerable for parenteral dose of the drug [5]. Nanosuspensions’ manufacturing involves the production of an enormous number of small particles with larger surface area. Owing to high interfacial tension, there is significant increase in the free energy of the system, which makes the system thermodynamically unstable. Therefore, nanoparticles will undergo agglomeration to reduce the free energy of the system. Incorporation of stabilizers to the nanosuspension system such as surfactants and polymers can reduce the interfacial tension between the nanoparticles and dispersion medium. Firstly, stabilizers act as wetting agents and reduce the interfacial tension between the nanoparticles and the dispersion medium. Secondly, stabilizers provide a barrier for the drug particles against agglomeration [20]. The amount and type of stabilizer used has a critical effect on physical stability as well as on the in-vivo performance of the nanosuspension system. Commonly used stabilizers are poloxamers, polysorbates, lecithin, povidones, etc. In addition, the choice of organic solvents and co-surfactants is important to formulate nanosuspensions when using submicron emulsions as templates. Moreover, characteristics of the active drug moiety and preferred route of administration influence the addition of other additives such as salts, buffers, osmogent, cryoprotectants etc. [5].

### 2.4. FDA Perspective on Excipients

Excipients are inactive ingredients that are intentionally added to pharmaceutical dosage forms and are not intended to exert a therapeutic effect [21]. Most of the formulations contain a small percentage of the active drug and typical major components are additives. Due to safety reasons, there are limitations on the type and quantity of an additive to be included in an injectable drug formulation. Commonly used excipients in the formulation of submicron emulsion/suspension are: buffering agents, tonicity agents, preservatives, wetting agents, complexing and dispersing agents, solvents and co-solvents, etc. [22]. Selection of excipients depends on toxicity issues, pharmaceutical acceptability of additives and excipients’ ability to withstand terminal sterilization and aseptic processing [18,22]. The choice of additives also depends on [22]:

1. Drug-excipient compatibility.
2. Compatibility of the excipient with manufacturing process and container–closure system.
3. Excipient impact on quality, safety, and effectiveness of the drug product.
4. Route of administration.
5. Dose volume and intended use of the drug product: single versus multiple dose.

IPEC (International Pharmaceutical Excipients Council) has classified excipients into the following three classes based on the availability of safety information:

- New chemical excipients: A full safety evaluation program is required for these excipients. A drug master file (DMF) must be filed with the FDA for a new excipient. The DMF contains relevant safety information.
- Existing chemical excipients—first use in man: Animal safety data are available for this class of excipients. Additional safety information is required when there is a change in dosage form, route of administration, higher dose, etc.
- New modifications or combinations of existing excipients: This class of excipients indicate a physical reaction and not a chemical reaction. Thus, no additional safety evaluation is necessary.

The FDA has published a list of substances in the Code of Federal Regulations that are generally regarded as safe (GRAS). Besides, the FDA maintains a list of inactive ingredients entitled the inactive ingredient guide (IIG) that are approved and can be incorporated into drug products. The IIG mentions a list of the maximum amount of excipients that can be incorporated in to drug products intended for a specific route of administration. Excipients are an integral part of drug formulation; however, the FDA does not currently have any process to separately evaluate the safety of the inactive ingredients. Rather, the excipients are reviewed and approved as “components” of drug/biologic product in the application. This regulatory process is suitable from a scientific viewpoint [23]. Table 1 below mentions a list of some commonly used excipients approved by the FDA (https://www.accessdata.fda.gov/scripts/cder/iig/index.Cfm) and the maximum allowable amount per unit dose used in formulation of colloidal drug delivery systems (DDS) as per the Inactive Ingredient Guide (IIG):

### 2.5. Physico-Chemical Barriers in Development of Submicron Drug Delivery Systems

Despite the advancements in nanotechnology, instability of biphasic systems is one of the shortcomings for its application to pharmaceutical industry. Submicron emulsion instability is caused by mechanisms such as flocculation, creaming, coalescence and Ostwald ripening [24]. Flocculation is the aggregation of two or more droplets without the loss of individual identity of the droplets. pH and also ionic strength of the polar phase affect flocculation. Density difference between the dispersed phase and dispersion phase leads to creaming. The creaming rate depends on the particle size and volume fraction of the dispersed phase. Coalescence is defined as a process in which there is collision of two or more droplets to form a larger droplet. Ostwald ripening occurs because of polydispersed droplets [6]. Instability issues such as flocculation, creaming and aggregation can be reduced by nanosizing of the droplets as well as usage of nonionic surfactant. Ostwald ripening leads to large droplet size distribution and turbidity of the nano scale emulsion. Elasticity of the droplets can be increased by the addition of a polymeric surfactant to reduce Ostwald ripening [25]. The storage stability of the nano-suspension system is critical as aggregation (agglomeration of two or more drug particles) and sedimentation are two common instability issues associated with nano-suspensions. When gravitational force exceeds the buoyancy force of the dispersion system, sedimentation occurs. Further, owing to the high energy of the particles in the amorphous state, particles may transform to crystalline form, which possesses low free energy. Besides the above-named physical instability aspects, hydrolysis and oxidation are perturbing and troublesome aspects of chemical instability in nano-dispersion systems [26,27].

## 3. Scientific and Regulatory Considerations for Approval of Biphasic Colloidal Systems

In the discovery phase of pharmaceutical formulation development, after the identification of a new chemical/molecular entity (NCE/NME), there is a necessity to develop a vehicle to assess the toxicity or activity in in vitro and in vivo evaluations [28]. From a regulatory perspective, safety, efficacy, and quality concerns are the key barriers that are encountered during non-clinical and clinical studies for a successful launch of the dosage form. Overall stability coupled with absence of immunological reactions to the used excipients must be demonstrated [23]. Under the FDC (Food Drug and Cosmetic) Act Section 505, there are two regulatory pathways under which an NDA (New Drug Application) can be submitted to the FDA for review, namely 505(b)(1) and 505(b)(2). The 505(b)(1) route contains comprehensive safety and effectiveness data for an NCE (new chemical entity) that has never been approved previously. Submission consists of data on non-clinical/pre-clinical and clinical trials, and pharmacokinetic and pharmacodynamic results. NDA submission under the 505(b)(2) pathway is granted for significant changes for already approved drugs, such as new formulations, strengths, dosage forms, dosing regimens, routes of administration, combination products of two already approved products, monograph deviations and substitutions of an active ingredient in a combination product [29].

Generic copies of the branded nanomedicines product are submitted for review to the FDA under 505(j). The submission for generic versions is called an Abbreviated New Drug Application (ANDA). The proposed generic version contains the same active ingredient, strength, dosage form, administration route, quality, labeling, performance features and intended use to a previously approved reference listed drug (RLD) [29]. The usage of generic products extended to 88% of prescription drug use in the US after the Hatch–Waxman Act came into force in 1984. Generic drugs should be pharmaceutically equivalent to the reference listed drug and show bioequivalence (BE) when administered to patients under specified labeling conditions of the RLD. A generic copy should also demonstrate therapeutic equivalence along with pharmaceutical equivalence and bioequivalence. In the past few decades, the formulation development of nanosized medicine has been applied immensely to parenteral dosage forms to enhance drug delivery. Stringent equivalence standard requirements are essential due to the complexity of nanoscale medicines. As a consequence, there have been challenges in establishing active moiety sameness, physico-chemical equivalence in drug products and in-vivo drug exposure profile equivalence between innovator and generic drug products [30].

The overall regulatory paradigm recommends a generic version of parenteral nanomedicines to be qualitatively (Q1) and quantitatively (Q2) the same as that of the reference listed drug. The exceptions are excipients such as preservatives, antioxidants, and buffers, provided that the differences do not impact the safety and efficacy profile of the drug product. Additionally, to ensure therapeutic equivalence between the generic copy and the RLD, a two-pronged approach is generally applied:

1. Comprehensive comparison of physico-chemical characterization of at least three batches of test and reference products.
2. In-vivo studies to demonstrate BE.

The physiochemical characterization is designed to show drug substance sameness, formulation Q1/Q2 sameness, structure, particle size distribution, in-vitro drug release (Q3) as well as stability [30].

### 3.1. Critical Quality Attributes of Colloidal Drug Delivery Systems

#### 3.1.1. Particle Size Distribution

Particle size distribution (PSD) governs the physical, chemical and biological properties as well as the clinical outcome of the nanomedicines. Analogous to all parenteral dosage forms, IV emulsions are also required to meet pharmacopeial specifications. Parenteral emulsions must be sterile, non-pyrogenic, biodegradable, isotonic, non-toxic and physically and chemically stable. Further, the droplet size must be <1 µm and typically ranges from 100 to 500 nm [31]. Although there are various PSD techniques, the FDA has not identified which technique is the most appropriate for PSD characterization. “Single particle”and “ensemble” methods are used for particle size determination. Ensemble methods detect a signal generated by numerous size ranges. The signal is deconvoluted (inverted) and assumes gaussian particle distribution. As opposed to ensemble methods, single particle methods detect a response given by a single particle. Single-particle methods require sufficient dilution so that single particle passes through optic region of the equipment whereas ensemble methods do not require much dilution. Different PSD techniques generate response based on volume, number, surface area, weight or intensity. Dynamic light scattering (DLS), a widely used particle size measurement technique, generates signal based on intensity, whereas the laser diffraction technique generates signal based on volume. Full descriptions of coarse and fine particles can be found in the regulatory body data on D10, D50 and D90. D10 describes the 10th percentile, D50 describes the median and D90 describes the 90th percentile of the particles that fall under a specific size [32]. The width of the distribution or SPAN value is given by (D90-D10)/D50. The homogeneity/heterogeneity of the distribution is further explained with the polydispersity index, which varies from 0.0 to 1.0. The higher the number, the higher the heterogeneity of the particle distribution. Further, the sponsor needs to demonstrate the reproducibility of the method by showing data from multiple batches [29].

#### 3.1.2. In-vitro Dissolution Test

To ensure the quality of the product and, therefore, its safety and efficacy, the sponsor must develop and validate a discriminatory in-vitro dissolution test. Oftentimes, the FDA recommends an in-vitro approach for generic approval between test and brand products when clinical studies are not feasible. One of the tests in the in-vitro approach is a discriminatory in-vitro release test. The choice of release medium, volume, apparatus, agitation and temperature will affect the release of complex nanomedicines [29]. The reported methods for the in-vitro release test are continuous flow methods, sample and separation methods and membrane diffusion methods [33]. The drug release data are studied using various models such as the zero-order model, first-order model, Higuchi model, Korsemeyer–Peppas model, etc., to understand the drug release mechanisms [34].

#### 3.1.3. Amorphous/Crystalline Content

For clinical settings to be unchanged, nanomedicines should remain stable throughout shelf life so that the amorphous/crystalline content does not change. The amorphous/crystalline ratio should be part of regulatory specifications. A method that can quantify the lowest amount of amorphous or crystalline substance must be developed and validated. The reported methods include solid-state NMR and thermal and X-ray diffraction [29]. Additionally, other physical parameters such as charge and shape impact in vivo performance. The charge of any drug delivery system is a vital characteristic that governs its stability in suspension, due to electrostatic interactions, as well as its performance in vivo [13].

#### 3.1.4. Rheology and Sterility

Viscosity measurement provides information on the influence of colloidal systems on drug release [35]. Besides, sedimentation characteristics during storage are significantly important as non-uniform distributions of drug can lead to failure due to over-dosage. In addition to aforementioned factors, another critical factor to be considered for nanosystem i.v. delivery is syringeability. The syringeability is a measure of the pressure associated with injection with a needle of specific gauge and length [33]. In addition, it is important for a formulation to be sterile for its safe application for treatment. It has been demonstrated that bacterial spores can be entrapped during the process of crystallization and that these spores are resistant to moist, dry and chemical sterilization [33]. Moist heat sterilization techniques such as autoclaving are unsuitable for submicron biphasic systems as they involve higher temperature and pressure settings. Sub-micron biphasic systems are usually sterilized by aseptic sterilization techniques without altering their physico-chemical properties [36].

## 4. Applications of Colloidal Carriers

### 4.1. Total Parenteral Nutrition

Energy deficit is a common problem among ICU patients. Parenteral nutrition (PN) can improve caloric delivery to critically ill patients whether used for the short-term or the long-term. In artificial nutrition, lipids are an important source of calories. ILEs (intravenous lipid emulsions) are one of the vital components of PN regimen as they provide a dense source of energy, as well as essential and conditionally essential fatty acids. Commercially available ILEs are complex mixtures of oil-in-water. Emulsification allows the lipid phase and aqueous phase to co-exist at a lower surface tension as a homogeneous dispersion of fat globules in water. ILEs contain thousands of fat globules per mL, with a mean diameter of ≈0.25–0.5 μm. ILEs differ from each other in terms of oil source, fatty acid composition, lipid concentration and other ingredients such as vitamins. Two common ILE formulation delivery systems are the 2-in-1 system, with two macro-nutrients (glucose, amino acids) and all micronutrients in a single bag (ILE separate), and the 3-in-1 system (total nutrient admixture), with three macronutrients and all micronutrients in a single bag. Thus, PN alone or in combination with enteral nutrition (EN) can improve caloric delivery to all critically ill patients [37,38].

### 4.2. Vaccine Delivery

Vaccination is a remarkable means of prevention of infectious diseases thereby contributing significantly to an increase in life expectancy. Despite these exceptional achievements, there is still an on-going requirement to improve vaccine delivery in order to combat infectious diseases. Currently, most vaccines are administered via invasive routes. Parenteral route of vaccine administration can trigger systemic immune response [39]. Inability of vaccine candidates to invoke suitable immune responses leads to failed vaccine development [40]. There is a necessity for the development of potent as well as safe adjuvants to deliver new generations of vaccines against infectious (e.g., pneumonia) and non-infectious (e.g., cancer) diseases [41,42]. The invention of Baker et al. provides composition and methods to stimulate immune responses using nanoemulsion and an inactivated pathogen via mucosal delivery [43]. Squalene *o*/*w* emulsion containing influenza vaccine was approved in Italy in 1997 [39].

### 4.3. Long-Acting Injectable (LAI) Therapy

Long-acting injectable formulations help sustain the therapeutic action of drugs in the body over desired time intervals. There is an increased frequency of administration of drugs that are susceptible to rapid in vivo clearance leading to poor patient compliance. Thus, development of controlled release strategies allows extended systemic exposure on administration of a single dose [44]. Key factors affecting drug release kinetics are variability in structure of the tissue, physiology of the recipient, rate of injection and technology format [45]. LAIs are administered in proximity of the affected tissue directing drug exposure over prolonged periods of time. LAI technology platforms include: (i) microencapsulation, (ii) in-situ forming depots (gels/implants) and (iii) molecular and particulate delivery systems. Invega Trinza^®^ and Invega Sustenna^®^ are sterile nanosuspensions of paliperidone palmitate that were first registered with a dose of 150 mg/human monthly and 525 mg/human every three months, respectively [44].

### 4.4. Anti-Cancer Drugs and Diagnostic Agents

Conventional chemotherapeutic agents to diagnose and treat cancer attack tumor cells and healthy body cells non-specifically, leading to life-threatening side effects. Encapsulation of these agents in the nanoparticle matrix as nanosuspensions has shown encouraging results in the specific targeting of anti-cancer drugs and diagnostic agents for the targeting of cancer cells [46]. In addition, many of the currently used anti-cancer drugs have low aqueous solubility and require use of toxic co-solvents such as cremophor to aid solubility. Development of such anti-cancer drugs as nanosuspensions avoids the use of toxic solvents to enhance solubility and use of biodegradable polymers greatly enhances safety profile of these drug loaded nanoparticle systems, Table 2 [10].

## 5. Conclusions

The sub-micron and nano drug delivery colloidal systems (emulsions and suspensions) described in this article revealed their immense potential for delivery of large and small organic molecules, long acting injectables, total parenteral nutrition, anti-cancer drugs and diagnostic agents. Complete characterization of these systems including particle size distribution, in-vitro dissolution, amorphous/crystalline content, rheology and sterility is essential to gain an understanding of how effectively the system meets clinical needs and receives regulatory agencies’ approval. These systems are well known, and many successful marketed drug formulations are in current use. With advancements in technology, these biphasic systems will become more mainstream as drug delivery systems of choice for the delivery of “hard to solubilize” and “hard to develop” injectable products.

## Figures and Tables

**Table 1:** List of commonly used excipients in formulation of colloidal DDS.

Excipient	Function	Route of Administration	Maximum Potency Per Unit Dose
Chlorobutanol	Antimicrobial preservative	Parenteral	0.5% *w*/*v*
Methylparaben	Antimicrobial preservative	IV	5% *w*/*v*
Bisulfite sodium	Antioxidant	IV	50 mg
Polyethyleneglycol 300	Co-solvent	IM	50% *w*/*v*
Disodium EDTA	Chelating agent	IM	10% *w*/*v*
Sorbitan monolaurate	Surfactant	IM	0.38% *w*/*v*
Castor oil	Lipid phase	IM	30% *w*/*v*
Monothioglycerol	Tonicity modifier	IM	0.5% *w*/*v*

**Table 2:** Over-view of parenteral nanomedicines approved by USFDA (https://www.accessdata.fda.gov/scripts/cder/daf/).

Dosage Form	Sponsor	Indication	Active Ingredient	Route of Administration
Paclitaxel Injection, USP	Grand Pharma Ltd.	Anti-tumor	Paclitaxel	Intravenous
Diprivan^®^	Fresenius Kabi USA LLC	General anesthetic and sedation drug	Propofol	Intravenous
ZYPREXA^®^ Intramuscular Injection	Lilly	Treatment of Schizophrenia	Olanzapine	Intramuscular
Phytonadione Injection, USP	International Medication Systems Ltd.	Treatment of Hypoprothrombinemia	Phytonadione	Intravenous/Intramuscular
Abraxane^®^ for Injectable Suspension, USP	Abraxis Bioscience	Treatment of Metastatic Breast Cancer	Paclitaxel	Intravenous
Invega sustenna^®^	Janssen Pharms	Antipyschotic	Paliperidone Palmitate	Intramuscular
Methylprednisolone Acetate Injectable Suspension, USP	Sandoz Inc.	Anti-inflammatory	Methylprednisolone Acetate	Intramuscular, Intraarticular, Intralesional
